# A website for pilot and feasibility studies: giving your research the best chance of success

**DOI:** 10.1186/s40814-019-0522-6

**Published:** 2019-11-05

**Authors:** Claire Louise Chan

**Affiliations:** Centre for Primary Care and Public Health, Queen Mary University of London, Yvonne Carter Building, 58 Turner Street, London, E1 2AB UK

## Abstract

This editorial introduces a website for pilot and feasibility studies. Pilot and feasibility studies are about giving research the best chance of success, but must be performed well to have the greatest benefit. The website was developed by the Pilot and Feasibility Studies collaboration who developed the CONSORT Extension to Pilot and Feasibility Trials as a resource to help triallists and researchers perform well conducted pilot and feasibility studies. The website is also aimed at those interested in the latest methodology for these studies. We aim to keep the site updated with the latest publications and events related to pilot and feasibility studies and welcome feedback and suggestions from the research community on further resources or events to add.

## Introduction

As an Associate Editor of *Pilot and Feasibility Studies*, it is clear from handling some of the submissions that there remains a need for improved conduct and reporting of pilot and feasibility studies. To address this need, and as a member of the Pilot and Feasibility Studies (PAFS) collaboration (see the ‘Acknowledgements’ section), who developed the CONSORT Extension to Pilot and Feasibility Trials [1, 2], we have designed a website to support those conducting pilot and feasibility studies and those carrying out methodological research on these types of studies. To our knowledge, there is no other similar online resource. The purpose of this editorial is to draw attention to the website. I briefly describe how the website was developed, introduce the content included in the website, and finally discuss plans for maintenance and development of the website as a resource for the research community and to which they can contribute.

### What are pilot and feasibility studies?

There are an increasing number of studies referred to as pilot and feasibility studies. A feasibility study explores the question of whether something can be done, whether we should proceed with it, and if so, how. Pilot studies are a subset of feasibility studies and ask the same questions as feasibility studies but are additionally conducting part (or all) of a future study on a smaller scale. These definitions were the result of recent extensive research into the use of these terms [3].

### Why perform pilot and feasibility studies?

The purpose of performing pilot and feasibility studies is to clarify any uncertainty about the feasibility of conducting a future study. Each study will have its own areas of uncertainties about feasibility. Some examples might include the feasibility of recruiting and retaining a sufficient number of patients, logistics of randomisation, testing the study protocol procedures, feasibility of data collection and which outcomes to use, and feasibility of implementing the intervention [4–6]. Findings from the pilot or feasibility study inform the design of the future study. Solutions can be found to problems encountered during the pilot or feasibility study, meaning that the research team are less likely to encounter problems in the definitive study and successful delivery is more likely. This is particularly important because main studies often require substantial time and resources, and there is increasing impetus to reduce research waste [7].

### What is the current state of conduct and reporting?

Despite the importance of pilot and feasibility studies, and the progress made to date, their conduct and reporting still require improvement. For example, many authors conduct hypothesis tests for effectiveness, even though pilot and feasibility studies are not formally powered to assess potential effectiveness [5, 8]. Moreover, authors sometimes incorrectly use the effect size determined in a pilot study to power their main study [9, 10]. Ideally, authors should pre-specify progression criteria to indicate whether to proceed with the main trial, but many studies do not report any such criteria [11].

Reporting of pilot and feasibility studies requires improvement, as shown by several recent reviews [11–13]. For example, authors of pilot studies are not reporting their reason for the pilot well enough; reports should include a clear list of feasibility objectives, the rationale for sample size, and progression criteria for continuing to the main trial [11]. Some studies are currently incorrectly being labelled as ‘pilot’ or ‘feasibility’ studies when in fact they are small underpowered effectiveness studies [14]. In reporting of pilot and feasibility studies, it is important to show how the findings have informed the future study and to assist readers preparing for similar future studies. This is important whether the study leads to a full-scale trial or not and may be particularly important if the study failed so that other researchers can learn from what did and did not work and consider potential alternatives [15].

## Why a website?

The idea for the website emerged as part of the on-going PAFS initiative [1, 2]. The call for improved conduct and reporting of pilot and feasibility studies has been addressed through several research papers, either highlighting the issue or providing advice and guidance [1–6]. However, we were not aware of anywhere where all the research on pilot and feasibility studies had been brought together and collated to provide an easily accessible resource for those conducting such studies. We first discussed the idea in March 2017 at one of the PAFS collaboration’s regular teleconferences and agreed the website would be funded through Sandra Eldridge’s NIHR Senior Investigator Award.

### How was the website created?

The first outline of the structure of the website was created in April 2017, and members of the PAFS collaboration were allocated different sections to populate. Draft versions of the website were shared and updated via a series of emails and teleconferences. A web designer was employed in April 2018 to create the website based on the final draft of the contents.

In May 2018, an email invitation was sent out to stakeholders previously involved in the consensus meeting during the development of the CONSORT extension for pilot trials. Individuals were invited to review the website before it went live. Those who agreed were sent a link and password to the website at the end of June 2018 and a review sheet to complete. Feedback was received and the website updated. The site went live in September 2018.

### What can I find on the website?

The website can be viewed at https://pilotandfeasibilitystudies.qmul.ac.uk/. The website comprises six pages: homepage, introduction page, resources page, noticeboard, who we are, and a contact page. The introduction page describes what pilot and feasibility studies are, including the distinction between internal and external pilot studies, provides examples of pilot and feasibility studies, reasons to perform such a study, and information on incorrect use of the terms pilot and feasibility. The resources page is divided into sections on design, analysis, and reporting, with further subsections within each of these. Resources are provided in the form of hyperlinks to published papers, with brief descriptive paragraphs as appropriate. The noticeboard page features news items, announcements, events, and courses concerned wholly or partly with pilot or feasibility studies, which might be of interest for people to attend, as well as details of past events and courses.

### Who is the website for?

The website is aimed at anyone conducting a pilot or feasibility study (for example, triallists and health services researchers, clinicians, health professionals, grant funding bodies, peer reviewers, journal editors) as well as those carrying out methodological research (for example, researchers, statisticians, students). For those interested in conducting a pilot or feasibility study, there are helpful resources on how to choose the objectives for a study, advice on choosing the study design and sample size to match the chosen objectives, and resources to guide appropriate analysis and reporting of the results. For those wanting to carry out methodological research, there is information on the latest publications and guidelines related to pilot and feasibility studies, as well as information about other relevant events such as workshops and training courses.

## How can I get involved?

We aim to keep the site updated with the latest publications and guidelines, as well as discussions and events related to pilot and feasibility studies. Minor updates are currently performed throughout the year, together with a formal annual review and update. We recognise that the website is unlikely ever to contain an exhaustive list of resources and encourage the research community to contribute additional material and relevant links using the ‘Contact us’ page on the website or sending an email to: pilotandfeasibilitystudies@qmul.ac.uk. We also welcome ideas for news items or events to add to our noticeboard.

## Conclusion

Pilot and feasibility studies, if conducted well, are important for increasing the chance of successful delivery of the future study. However, current conduct of such studies is suboptimal. We have therefore developed a website providing resources to help researchers conduct a pilot or feasibility study. The website is designed to support those conducting pilot and feasibility studies using randomised and non-randomised designs. Moreover, those carrying out methodological research on these types of studies can also benefit from using the site. We aim to keep the site updated with the latest resources and events related to pilot and feasibility studies. We welcome feedback and suggestions from the research community on further resources that would be helpful to include, as well as items to add to our noticeboard.

## Data Availability

Not applicable.
